# The gut microbiota and colorectal surgery outcomes: facts or hype? A narrative review

**DOI:** 10.1186/s12893-021-01087-5

**Published:** 2021-02-12

**Authors:** Annamaria Agnes, Caterina Puccioni, Domenico D’Ugo, Antonio Gasbarrini, Alberto Biondi, Roberto Persiani

**Affiliations:** 1 Università Cattolica del Sacro Cuore, Largo F. Vito n.1, 00168 Rome, Italy; 2 Dipartimento Di Scienze Mediche E Chirurgiche, Fondazione Policlinico Universitario Agostino Gemelli IRCCS, Largo A. Gemelli n. 8, 00168 Rome, Italy

**Keywords:** Microbiota; colorectal surgery; anastomotic leak, Surgical site infections, Oral antibiotic preparation

## Abstract

**Background:**

The gut microbiota (GM) has been proposed as one of the main determinants of colorectal surgery complications and theorized as the “missing factor” that could explain still poorly understood complications. Herein, we investigate this theory and report the current evidence on the role of the GM in colorectal surgery.

**Methods:**

We first present the findings associating the role of the GM with the physiological response to surgery. Second, the change in GM composition during and after surgery and its association with colorectal surgery complications (ileus, adhesions, surgical-site infections, anastomotic leak, and diversion colitis) are reviewed. Finally, we present the findings linking GM science to the application of the enhanced recovery after surgery (ERAS) protocol, for the use of oral antibiotics with mechanical bowel preparation and for the administration of probiotics/synbiotics.

**Results:**

According to preclinical and translational evidence, the GM is capable of influencing colorectal surgery outcomes. Clinical evidence supports the application of an ERAS protocol and the preoperative administration of multistrain probiotics/synbiotics. GM manipulation with oral antibiotics with mechanical bowel preparation still has uncertain benefits in right-sided colic resection but is very promising for left-sided colic resection.

**Conclusions:**

The GM may be a determinant of colorectal surgery outcomes. There is an emerging need to implement translational research on the topic. Future clinical studies should clarify the composition of preoperative and postoperative GM and the impact of the GM on different colorectal surgery complications and should assess the validity of GM-targeted measures in effectively reducing complications for all colorectal surgery locations.

## Background

Colorectal surgery represents the standard treatment for colorectal cancer in the context of a multimodal treatment that is administered according to the stage and localization of the disease [1–3]. Colorectal surgery is also the mainstay of treatment for diverticular disease [4], inflammatory bowel disease (IBD), mechanical bowel obstruction, some proctological disorders and acute conditions such as colic injury and ischemia [5]. The control of risk factors for complications after colorectal surgery has been actively pursued throughout the years. Many short- and long-term outcomes have been considerably ameliorated by the standardization of the surgical procedures and centralization to high-volume hospitals [6, 7]. Nevertheless, a substantial number of patients undergoing colorectal surgery experience postoperative morbidity, mainly in terms of postoperative ileus (POI) (10–30%), surgical-site infections (SSIs) (6.5–20%), anastomotic leak (AL) (2.7–20%) and re-admission (8.1–11.8%) [8–13].

There are well-recognized risk factors for postoperative complications, some of which are patient-related and unmodifiable (i.e., age, pre-existing comorbidities). Moreover, surgical complications have been associated with inadequate antisepsis, defects in the surgical technique and/or an insufficient learning curve for the specific colorectal procedure [8,^ 14^–17]. However, the occurrence of postoperative complications is not completely understood for all patients, as they occur even in patients with few risk factors [8, 18].

In recent years, a novel factor has been recognized among those involved in the physiology of the gastrointestinal tract and the entire organism. The intestinal microbial composition, or the gut microbiota (GM), is involved in the regulation of enterocyte wellness and gastrointestinal homeostasis. Additionally, gut microbial dysbiosis has been proven to play a significant role in the onset of disorders such as obesity, IBD, autoimmune diseases, diabetes, predisposition to infections and cancer [19, 20]. Last, a biunivocal relation of microbiota between trauma and stress (including surgical stress) has been identified [18]. Recently, these discoveries led to a challenge of the traditional mindset on the risk factors for surgical complications, proposing the GM as one of the determinant factors for their occurrence [18].

A better understanding of the complex interaction among the GM, colorectal surgery and perioperative care could significantly improve patient management, allowing for the optimization of surgical outcomes. In this review, we approach the knowledge on the GM from a surgical point of view, and we synthesize the current knowledge on the role of microbiota in colorectal surgery.

## Methods

The review of the literature was conducted with the following method:

- A search was conducted on Pubmed for all articles published up to May, 2020 with the following terms associated to “colorectal surgery” OR “hemicolectomy” OR “rectal resection” OR “colorectal cancer” OR “colorectal surgery complications” OR “surgery”: “microbiota” OR “microbiome”.
- The abstract was screened by two authors (AA and CP) and the articles selected from the abstract were evaluated in full text.
- After evaluation of the full text, the articles were included according to their pertinence in regards of the main topics of the article: physiologic response to surgical stress and healing after surgery, colorectal surgery complications, role of bowel preparation and role of probiotics/prebiotics.
- The reference list of the articles evaluated in full text was screened for any other relevant article and those articles were evaluated according to the same criteria.

### The GM and the microbiome: definition and physiology

The microbiota is defined as the totality of microorganisms that colonize a specific setting in a specific period. The human microbiota is the combination of all microorganisms that live symbiotically with humans (bacteria, fungi, viruses, archaea and protozoans). Most of these microorganisms are concentrated in the gastrointestinal tract and represent the GM. The GM is affected, in terms of composition and gene expression, by multiple environmental agents, including dietary factors, smoking, medicinal agents, stress, exercise, age, hormones and geographic location [21]. All these factors contribute to GM fluctuations, which have been recorded even on a daily basis, even though the GM has also shown an intrinsic tendency to return to a stable baseline [22]. The GM also has wide interpersonal variability [23]. For these reasons, a general consensus on the composition and characteristics of a “normal microbiota” has not yet been reached [24].

Another main factor that limits the study of the GM is that only 50% of the organisms in the human gut are cultivable in vitro [25]. Only in recent years, with the widespread use of techniques such as transcriptomics, proteomics and metabolomics, has a better understanding of GM composition, gene expression and function been reached [23]. The main techniques in use are the sequencing of the bacterial 16S ribosomal RNA (rRNA) gene, which is present in all bacteria and archaea and contains nine highly variable regions, allowing the different species to be easily distinguished [23], and random sequencing (shotgun metagenomic data), in which sequences are matched to those of known functional genes in databases, giving better insight into the functional properties of the GM. These methods are expected to be integrated in future years with mRNA, protein and metabolite profiling, which will further investigate the functional properties of the sequenced genes [22].

The sequencing of the human gut microbiome has enabled the identification of resident species that are usually constant, even though their relative percentages have wide variations among different individuals [22]. In a healthy individual, the GM is usually composed of one archaean phylum (*Euryarchaeota)* and five main bacterial phyla: *Bacteroidetes*, *Firmicutes*, *Actinobacteria*, *Proteobacteria* (including *Escherichia coli* and *Pseudomonas aeruginosa*) and *Verrucomicrobia* [26]. Microorganisms that feed on unabsorbed and undigested nutrients are those that seem to have an advantage in terms of survival. Accordingly, a significant effect of diet on the GM has also been identified. The Western diet, rich in sugars and animal fats and low in fiber, has been related to an increase in *Bacteroides* and a reduction in *Firmicutes*, while an increase in *Firmicutes* has been associated with a high-fiber diet [26–28].

To obtain a better comprehension of how the human host and GM actively interact and how alterations in the GM can be related to different diseases, the role of in vivo and translational research based on animal models has also been fundamental. The main strategies in use have been the manipulation of the host’s genetic background (gene knockouts); the manipulation of GM composition through controlled inoculation in germ-free (GF) or gnotobiotic mice; and ecosystem interventions, including dietary changes, antibiotic treatment and fecal transplantation. Through human GM inoculation into a GF mouse, it has been possible to obtain a reliable human GM model in which 100% of the phyla and 88% of the taxa were reproduced [29].

According to the results obtained with these methods, two types of GM have been distinguished by location: the mucosal-associated microbiota (MAM) and the luminal microbiota (LM) [30]. In humans, the LM is currently the most analyzed due to the simplicity of fecal sample collection. By contrast, the MAM is usually sampled using bowel-tissue biopsies obtained through endoscopy. In the MAM, *Bacteroidetes* and *Proteobacteria* are more represented, while *Firmicutes* and *Actinobacteria* are more abundant in the LM [30]. Although the implications related to the different compositions of the MAM and the LM are still poorly understood, the MAM is more permanent and less susceptible to external factors, such as diet or stress [31], and each bacterium found in the LM is found in the MAM, while some bacteria are exclusively present in the MAM. These differences are attributed to different antibacterial or mucosal factors, different oxygen tensions between the lumen and the mucosa and different access to nutrients [30]. The MAM is involved in the stimulation of mucus secretion and in the production of short-chain fatty acids (SCFAs), such as acetate, butyrate and propionate, which have been regarded as regulators of gut physiology and mediators of the host immune system. Butyrate, mainly produced by the *Firmicutes* phylum, is involved in colonocyte metabolism, enhances intestinal barrier function and mucosal immunity, and has anti-inflammatory and anticancer activity [23,^ 32^, 33]. Acetate, produced by anaerobes and in particular by *Bifidobacterium*, has been involved in defense mechanisms against external agents, such as infection by enterohaemorrhagic *Escherichia coli* (EHEC) [34].

### Physiological response to surgical stress and healing after colorectal surgery

Patients undergoing major surgery experience a complex endocrine and metabolic response to surgical stress, known as the catabolic phase. This phase is directly proportional to the severity of trauma and consists of significant modifications in carbohydrate and protein metabolism, together with altered hormonal production and responses [35]. Catabolic hormones, such as catecholamines and cortisol, increase, and the stimulation of the renin-angiotensin axis leads to fluid retention. The production of inflammatory cytokines, such as IL-6, is also involved [36]. Catabolic modifications usually develop in peripheral tissues such as muscle, fat and skin and aim to increase the supply of energy and protein substrates to improve tissue damage repair and wound healing and to preserve critical organ function. The catabolic phase usually reverts 3–8 days after surgery, and the patient transitions to an anabolic phase, characterized by a gradual restoration of body protein and fat stores and a final return to normal physiology [37]. The prolongation of the catabolic phase or its excessive magnitude may lead to counterproductive effects; therefore, most of the current perioperative strategies are targeted at minimizing these adverse effects [38].

Parallel to the systemic response to surgical stress, the process of wound healing occurs in all tissues that have been subjected to surgical trauma. This process has been principally studied in skin wounds, and it is divided into different phases. The first, associated with hemostasis, results in the activation of the inflammatory process. The subsequent inflammatory phase is characterized by neutrophil recruitment in an early phase, followed by monocyte and macrophage recruitment in a later phase. Macrophages are later involved in the resolution of the inflammatory process (through a switch from an M1 to an M2 phenotype) and in the initiation of the proliferative phase. This phase is characterized by the generation of new epithelial cells, by the migration/activation of fibroblasts and the consequent deposition of collagen, and through neoangiogenesis, all resulting in the formation of granulation tissue. The late phase is the remodeling or wound-maturation phase. During this phase, new, more resistant collagen is produced, and the scar is remodeled to acquire elasticity and resistance [39]. A scarless wound-repair process that usually occurs only in fetal healing [40] has been described in GF mice (mice without commensal microbiota) by Canasso et al. [40], who described that the wound-repair process of GF mice was faster than that of conventional mice and characterized by an earlier and greater presence of macrophages. When GF mice were recolonized with bacteria, the wound-repair process became analogous to that of conventional mice. In contrast, Okada et al. previously reported opposite results, finding that the tensile strength of the surgical wound in the earlier stages of wound repair (3rd to 8th postoperative day) was greater in conventional mice than in GF mice [41].

The anastomotic healing process has been modeled in the skin healing process, although it is less studied and still under investigation. The relative importance of the four layers of the bowel wall (mucosa, submucosa, muscularis propria and serosa) has still not been quantified [42]. Similar to skin wound healing, in the anastomotic healing process, the inflammatory phase is characterized by the activity of platelets, neutrophils, macrophages and fibroblasts, with the release of numerous growth factors and the activation of proteases with an increase in collagenolysis. Indeed, 48 h after surgery, a colorectal anastomosis loses 70% of its initial strength [43]. The following proliferative phase is characterized by smooth muscle cells and fibroblasts producing new collagen. During the remodeling phase, collagenase and other enzymes remodel the microscopic structure of the anastomosis, increasing its elasticity and contractile capacity [42–44]. The anastomotic healing process most likely begins at the level of the serosa, with the formation of a fibrotic cap that represents a matrix for fibroblasts [45]. The submucosal layer has been hypothesized to be the primary source of fibroblasts involved in the deposition of collagen [42]. The role of the mucosal layer has to be clarified, but it is probably related to the production of mucus, which may significantly contribute to anastomotic healing and to the regulation of bacterial translation [42]. Of note, previous studies detected no difference in the results between hand-sewn anastomoses, in which at least some of the distinctions between layers are often preserved, and stapled anastomoses, which involve all layers [46, 47]. AL has been attributed to derangement in one or more of the physiological processes involved (i.e., ischemia, excessive shift to a proinflammatory M1 macrophage phenotype in the inflammatory phase, or excessive production of collagen-degrading enzymes in the remodeling phase) [48]. From a clinical point of view, there are well-known risk factors for AL. Some are modifiable, such as obesity, nutritional status, preoperative bowel cleaning, preoperative anemia and intraoperative blood loss, whereas others, such as age, sex, comorbidities and radiochemotherapy, are unmodifiable [49, 50]. Of note, some of these factors have themselves been related to changes in microbiota composition [51].

### How does colorectal surgery affect the GM?

Several factors could be implicated in changes in the composition of the GM during and after colorectal surgery, including the following:

- Stress and the alteration of homeostasis: Stress and the GM are related through the bidirectional microbiota-gut-brain (MGB) axis, in which stress can influence the composition of the microbiota, and the microbiota can influence the host response to stress through immune, endocrine and metabolic pathways. The MGB axis has been extensively studied in trauma as well as in stress-related pathologies (i.e., posttraumatic stress disorder) [52]. Stress has been deemed able to modify the composition of the microbiota, and it may also be able to increase intestinal permeability and favor the translocation of GM microorganisms through corticotropin-mediated mechanisms [53]. In patients with major burns, 6 h after the injury, the GM undergoes important rearrangements, with a reduction in up to 90% of phyla such as *Bacteroides* and *Firmicutes* and a relative increase in *Proteobacteria* (*Escherichia coli, Pseudomonas aeruginosa, Enterococcus faecalis*) [54]. In critically ill patients, a direct correlation among a depletion in obligate anaerobes, an increase in pathogenic facultative anaerobes and the occurrence of major complications has been detected [55]. Finally, as demonstrated in a rat model, the MAM composition shifts after colectomy, with a significant increase in *Enterococcus, Escherichia* and/or *Shigella* spp. [56]. It is still unclear, however, to what degree the alteration of the MGB axis may be responsible for the detected shift in the GM in these situations.
- Exposure to oxygen: Many species in the GM are facultative anaerobes or obligate anaerobes. Exposure to oxygen during colorectal surgery (i.e., during bowel section or anastomosis) could cause a significant depletion of these species. In 2014, Shogan et al. demonstrated that the opening of the bowel caused a loss of “good” obligate anaerobes, such as some *Bacteroides*, and a gain of “bad” facultative anaerobes, such as *Enterococcus*, in a rat model. This change was detected in the MAM but not the LM [56].
- Tissue ischemia: Bowel and vascular sections may cause temporary or permanent ischemia of the neighboring gastrointestinal tract. Ischemia itself, or ischemia–reperfusion syndrome, can cause significant changes in the GM. In 2012, Wang et al. created an ischemia–reperfusion model in rats after 30 min of colic ischemia. They identified a change in the GM 1 h after reperfusion that reached a peak at 6 h after reperfusion and then gradually recovered; this change consisted of an early increase in *E. coli* and *Prevotella* and a later increase in *Lactobacillus*, in line with reperfusion and epithelial healing [57].
- Type of reconstruction: Different types of surgical reconstruction may lead to various consequences, such as a variation in the quality of food digestion and in the absorption of nutrients and vitamins. Food digestion and nutrient absorption have been related to the action of the GM [58], and it has been theorized that alterations in the GM due to surgical intervention and the type of bowel reconstruction may be partly responsible for malabsorption and/or maldigestion following certain surgical procedures (especially bariatric procedures) [18, 59].
- Chemo/radiotherapy: Patients undergoing colorectal surgery may have been exposed to neoadjuvant or induction therapy before the surgical procedure [1–3]. Previous studies have detected specific GM changes associated with the administration of specific chemotherapeutic agents [60]. Radiotherapy also seems to have a significant impact on the GM, as detected in some murine and human studies [61, 62]. Moreover, there is preliminary evidence that radiotherapy causes a phenotypic shift in certain bacterial species, increasing their virulence and tissue-destructive capacity [63].

The current hypothesis developed by some authors is that behind the occurrence of surgical complications (especially infective complications) is an impairment in the physiological return to basal GM homeostasis due to one or more of these factors. This delay could favor an increase in pathogenic bacteria and in the virulence of the commensal species and could even lead to immune system compromise or collapse [18].

### Surgical complications and the involvement of the GM

The most frequent complications after colorectal resection are POI, SSIs and AL. These complications have been regarded as the main causes of unplanned re-admission, as reported in a 2013 meta-analysis that related 33.4% of all re-admissions to bowel obstruction, 15.7% to SSIs and 12.6% to intraabdominal abscesses [64]. Patients undergoing diverting stoma and the exclusion of the distal colon are also known to develop diversion colitis, which may favor functional and infective problems after stoma closure [65–68].

#### Postoperative ileus

POI is defined as an absence of intestinal motility following the surgical procedure. The absence of postoperative peristalsis is considered “normal” for up to 24 h in the small intestine, up to 48 h in the stomach and up to 72 h in the large intestine. For this reason, POI is usually defined as an absence of intestinal function after the fifth postoperative day, accompanied by nausea and/or vomiting [69]. POI is reported in 10–30% of patients after abdominal surgery and is linked to an increased length of stay and an increased rate of reoperation [13, 70]. The occurrence of POI has been linked with different mechanisms, such as inflammation, inhibitory neural reflexes and neurohumoral peptides [13]. The GM is involved as a direct modulator of gut synapses that impair gastrointestinal motility or as a possible activator of dendritic cells, macrophages and monocytes involved in the mechanisms of inflammation [71, 72]. Dendritic cells produce IL-12, which is able to activate pathways that lead to POI. Moreover, IL-12 stimulates IFNɣ production by T helper 1 (Th1) cells, leading to the production of iNOS and NO by local macrophages, which contribute to the inhibition of smooth cell contraction [72]. In their study, Pohl et al. noted a substantial reduction in IL-12 and iNOS, implicated in POI, after the depletion of the GM with oral antibiotics (OABs). Notably, this reduction was maximal in the colon and moderate in the small intestine [72], possibly due to a different physiopathological mechanism between the inflammatory cells and the GM and the different locations, even though this consideration is highly hypothetical. Overall, there is still scarce evidence linking the role of the GM to POI, and further research is needed.

#### Postoperative adhesions

Postoperative adhesions are a frequent consequence of abdominal surgery, and they are reported in 63%–97% of patients after major abdominal procedures [73]. Postoperative adhesions are associated with obstructive complications that may occur in the immediate postoperative period or even months or years later. The incidence of small bowel obstruction due to peritoneal adhesions after abdominal surgery is 2.1%–4.6% [74, 75]. In the era of laparoscopy, the incidence of postoperative adhesions and the rate of re-admissions directly related to adhesions have been significantly reduced after colorectal surgery [76, 77]. However, postoperative adhesions still represent a significant burden and a cause for increased costs [77].

Adhesions occur as a consequence of peritoneal irritation due to surgical trauma or local infection, leading to a pathological healing mechanism that results in an imbalance between fibrin deposition and degradation. When this imbalance occurs, fibroblast recruitment, the production of the extracellular matrix (ECM), collagen deposition, and angiogenesis lead to the development of adhesions [73]. This is favored by inflammation, the production of cytokines and oxidative stress. The role of mast cells and their serotonin release also seem to be prominent [56,^ 78^, 79]. Recently, most of these mechanisms have been linked to the role of the GM [56]. In 2001, Bothin et al. compared the peritoneal adhesion process of GF rats, ex-GF rats and *E. coli*-monocontaminated or *Lactobacillus*-monocontaminated rats. They demonstrated that GF mice had a lower capacity to form peritoneal adhesions. When their microbiome was restored, the adhesion-forming ability was restored as well. In particular, *E. coli*-monocontaminated rats were more capable of creating peritoneal adhesion than rats contaminated by *Lactobacillus*. The authors stressed the probable role of bacterial leakage or translocation through the sutures to justify these results, supported by the well-known appearance of most adhesions in the area of bowel anastomosis [80]. Moreover, recent studies have consistently demonstrated that serotonin production is lower in GF mice and that it is probably mediated by SCFA production [81, 82].

#### Surgical-site Infections and Anastomotic Leak (Table 1)

**Table 1:** Preclinical and translational studies investigating the role of GM in determining AL

Author (year)	Sample size	Population	Group/groups	Outcomes	Results	Main findings/hypothesis
Cohn (1955) [1]	14 cases	Dogs	1. 8 dogs -antibiotic at the site of anastomosis and orally (achromycin) 2. 6 dogs – no topical antibiotics	Bowel viability after colic anastomoses with devascularization	1. 75% dogs had viable colon 2. 100% dogs in the control group died after ischemic perforation	Topic antibiotics help maintaining viability of the ischemic bowel and allow for regeneration
Abrams (1963) [2]	44 cases	Mice	1. Germ-free mice (24 cases) 2. Conventional mice (20 cases)	Status of the ileal mucosa and lamina propria	1. Lamina propria poor of vessels, lymphocites and mononuclear cells; small Peyer’s patches with low mitotic activity; thin mucosal layer 2. Lamina propria rich of lymphocites, mononuclear and plasma cells; well-represented Peyer’s patches with high mitotic activity; well-represented mucosal layer	GM promotes the trophism of the ileal mucosa
Schardey (1994) [3]	57 cases	Mice	1. Pseudomonas inoculation 2. Controls 3. Decontaminated	AL (esophagojejunal anastomosis)	AL rate: 1. 95% 2. 80% 3. 6%	Bacteria could play a major role in the pathogenesis of AL following gastrectomy
Okada (1999) [4]	49 cases	Mice	1. Germ-free, 2. Conventional 3. monocontamina ted with Lacto- bacillus acidophilus La5 4. Escherichia coli X7 5. ex-germ-free (repopulated)	Anastomotic healing (ileum and colon)	2. and 5. had higher anastomotic bursting pressure both in the ileum and in the colon compared with the other groups	The establishment of a normal intestinal flora restores the healing capacity of the anastomosis
Jones (2013) [5]	*NA*	Drosophila larvae, Mice	Germ-free drosophilia colonized with 6 different bacteria (3 g + and 3 g-); Germ-free wild-type mice vs Nox-1 deficient mice feeded with L. rhamnosus and E. Coli	Response of gut epithelia to different bacteria	Drosophilia’s gut colonized by L. Plantarum shows increased niche cells proliferation.; Germ-free WT mice feeded with L. Rhamnosus have an increased ROS production in the small intestine	Lactobacillus induces ROS production Bacteria-induced ROS production promotes epithelial proliferation and intestinal barrier functions
Van Praagh (2016) [6]	16 cases	Human tissue (donuts from colorectal surgery)	1. Patients who developed AL 2. Patients without AL	AL	Lachnospiraceae family in GM found on the doughnuts is significantly higher in the AL group	Correlation between Lachnospiraceae, BMI and anastomotic leak
Van Praagh (2019) [7]	123 cases	Human tissue (donuts from colorectal surgery)	1. Patients who developed AL 2. Patients without AL 3. Patients treated with C-seal 4. Patients not treated with C-seal	AL	3. High AL rates. No association between AL and GM 4. AL linked with low microbial diversity, high level of Bacteroidaceae and Lachnospiraceae families	Low microbial diversity of GM is linked with AL in patients not treated with C-Seal

SSIs are defined as infections that occur within 30 days after an operation, and they involve the skin and subcutaneous tissue of the incision (superficial incisional) and/or the deep soft tissue of the incision (deep incisional) and/or any part of the anatomy (for example, organs and spaces) other than the incision that was opened or manipulated during an operation (organ/space) [83]. SSIs have been reported in up to 30% of patients after colorectal surgery [84]. Incisional SSIs are the most frequent type of SSIs, described in 6.2%–21.6 of patients [85–87]. Many strategies have been applied to reduce the rate of incisional SSIs, including the administration of intravenous antibiotics, careful intraoperative bowel manipulation and the use of wound protectors. These strategies have proven effective, although there are still many incisional SSIs that are not preventable [84] and a subset of patients with less controllable risk factors (patients who are immunocompromised or are diabetic, patients undergoing emergency surgery, especially those with bowel perforation where the surgical field is highly contaminated) [88, 89]. The most represented pathogens in incisional SSIs are actually bowel bacteria, such as *E. coli*, *P. aeruginosa* and *Enterococcus* spp. [90]; therefore, the role of GM manipulation is under the spotlight to prevent this complication. Improved healing and outcomes have been correlated with increased bacterial diversity and an instability of the local microbiome composition [91]. Probiotics as *Lb. plantarum* have been related to beneficial local immunomodulatory effects and to interference with pathogen colonization by *P. aeruginosa*, *S. aureus*, and *S. epidermidis* [92–94]. Probiotics also exert an indirect beneficial effect on cutaneous health through effects on systemic immunity, enhanced nutrient absorption, and the modulation of the gut-brain-skin axis. The main mediator of the gut-brain-skin axis is the posterior hypophysis hormone oxytocin [95], the production of which is significantly increased by probiotics, resulting in increased wound healing capacity both in mice and in humans [96]*.*

Deep/organ-space SSIs have a reported incidence of 3.8%-11.5% after colorectal surgery [85–87]. The most frequent cause of organ/space SSIs, especially in rectal cancer surgery, is AL [97], even though it has been stressed how some underestimation of the AL rate has been associated with the deep/organ-space SSI definition [98]. AL has an incidence of 2–20% after colorectal surgery. Most ALs occur after low anterior rectal resection, especially when the anastomosis is performed < 7 cm from the anal margin [11, 49]. Some physiopathological studies have assessed the role of the GM in favoring anastomotic healing. Physiological levels of reactive oxygen species (ROS) promote epithelial proliferation and intestinal barrier functions [99], and human commensal *Lactobacilli* have been shown to induce ROS generation in intestinal epithelial cells [100]. Other studies demonstrated that specific GM species promote the re-epithelization of the injured mucosa by stimulating enhanced proliferation of intestinal epithelial cells in the nearby crypts [101, 102]. Accordingly, previous investigations on GF mice have demonstrated a reduced rate of intestinal epithelial cell migration [95, 101]. In 1999, Okada et al. compared the anastomotic bursting pressures of ileal and colic anastomoses performed in GF, ex-GF (conventionalized), conventional, *Lactobacillus*-monocontaminated or *Escherichia*-monocontaminated rats. They found higher anastomotic bursting pressures in conventionalized and conventional rats than in GF and monocontaminated rats [103]. One of the first studies suggesting a direct role of the GM in AL was carried out by Cohn and Rives in 1955. They performed colic anastomosis in dogs, creating a model for leakage through the systematic devascularization of 5 cm of the perianastomotic bowel. Following this procedure, the dogs were re-fed. One group of dogs was intra- and postoperatively treated with topical and systemic antibiotics (achromycin administered *per os* and at the anastomotic level through a small tube inserted during surgery and intramuscular penicillin), while the other group was treated with saline solution. The anastomoses of dogs treated with antibiotics healed, and ischemia subsided in 6/7 dogs, while AL caused by ischemia was uniformly fatal in 6/6 dogs treated with saline solution [104]. In 1994, Schardey et al. focused on esophagoduodenal AL after total gastrectomy. They created three rat models, one experimentally inoculated with *P. aeruginosa*, one conventional model and one decontaminated by OABs, and the rates of AL were 95%, 80% and 6%, respectively [105]. Some of these preliminary studies suggested that diversity in the microbiota was key to preventing AL, while others suggested that the key might in fact be local decontamination, even though antibiotic administration led to less diversity. This apparent paradox may be explained by the presence of disruptive species that are controlled either by decontamination or by microbial diversity that contains their excessive proliferation.

Following these preliminary findings, some studies focused on the composition of the GM in patients experiencing AL. In 2016, van Praagh et al. conducted a pilot study to assess the composition of the GM at the anastomosis level after rectal resection. They collected the "doughnuts" from the circular stapler used for anastomosis and extracted the 16S rDNA from 15 doughnuts: 8 from patients experiencing AL and 7 from patients with no AL. Patients who did not develop AL had higher microbial diversity, while patients who experienced AL had less microbial diversity and a greater representation of *Lachnospiraceae*, in particular of the mucin-degrading *Ruminococci spp*. [106]. These results were supplemented by the publication of an extended version of this analysis in 2019, which confirmed that AL was associated with low microbial diversity and with a high abundance of *Bacteroidaceae* and *Lachnospiraceae* (in particular *Blautia obeum*). In conclusion, the definition of protective and favorable preoperative GM “signatures” for AL was proposed [107]. In 2019, Palmisano et al. studied the composition of the preoperative fecal microbiota in patients undergoing surgery for colorectal cancer. Patients with AL had a relative paucity of *Faecalibacterium prausnitzii and Barnesiella intestinihominis* and a relative abundance of bacteria promoting dysbiosis, such as *Acinetobacter Iwoffii*, *Hafnia alvei* and *Acinetobacter johnsonii* [107].

Another concept investigated by some authors is the “phenotypic shift” of some bacterial species to a more virulent phenotype. In 2015, Shogan et al. described results obtained in a murine model in which colon resection with a perianastomotic segmental devascularization of 2 cm was performed. Fifty percent of the rats subjected to devascularization developed AL. At the microbiological assessment, the site of AL was colonized by bacteria with increased expression of collagenase activity. In particular, *E. faecalis* and *Proteus mirabilis* had the highest collagenase activity, and there were two types of *E. faecalis*, one with low collagenase activity (E1) and one with high collagenase activity (E2) [48]. E2 *E. faecalis* had different gene expression levels and showed a marked capacity to degrade collagen I and activate tissue MMP-9. Moreover, when a series of rats undergoing colorectal anastomoses were experimentally inoculated by enema, this bacterium was able to induce AL associated with the depletion of intestinal collagen. In another model of colorectal anastomoses where topical and parenteral antibiotics were introduced together with the devascularization of the anastomosis, the administration of topical antibiotics was capable of reducing the levels of MMP9 and eliminating the E2 strain [48]. In 2012, Oliveira et al. demonstrated a similar phenotypic shift (P1/P2) for *P. aeruginosa*. They analyzed four groups of rats: in group 1, the rats underwent colic resection with colic anastomosis; in group 2, in addition to undergoing resection and anastomosis, the rats were inoculated with *P. aeruginosa* at the level of the cecum; in group 3, rats were subjected to radiotherapy before resection and anastomosis; and in group 4, rats underwent radiotherapy, resection, anastomosis and *P. aeruginosa* inoculation. In group 4, *P. aeruginosa* acquired a single-nucleotide polymorphism mutation leading to the codification of a truncated protein. This mutation was imputed to radiation. The novel P2 phenotype was associated with pyocyanin production, increased collagenase activity, and high motility and displayed destructive activity even against cultured intestinal epithelial cells [63].

In conclusion, most of the recent studies identified low microbial diversity, the prevalence of Enterobacteriaceae and their virulence shift as the prevalent mechanism associated with leakage. This specific phenotypic shift may be triggered by radiotherapy. According to this hypothesis, colorectal anastomoses, which are actually those with the greater reported risk of AL, are also those where preoperative radiotherapy is more frequently applied for the treatment of rectal cancer [8, 63].

#### Diversion colitis

During surgery, a loop ileostomy is created to divert the fecal content to protect the colorectal anastomosis and diminish the risk for AL in patients with high-risk anastomoses (i.e., low colorectal anastomoses, anastomoses in patients who underwent preoperative chemoradiation) [108]. In patients with a diverting ileostomy, the residual colon is defunctionalized. The defunctionalization of the colon may cause the occurrence of diversion colitis, namely, the inflammation of excluded segments of the colon in patients who have undergone colostomy or ileostomy and have no history of IBD [108]. The prevalence of diversion colitis is extremely high, as it reaches almost the entire population of patients with excluded colons if the phenomenon is followed prospectively, beginning 3 to 36 months after ileostomy creation [109, 110]. Even if its prevalence accounts for almost 100% of patients, diversion colitis is symptomatic in only 30% of patients. Symptoms consist of abdominal discomfort, tenesmus, rectal bleeding and/or mucus discharge. Endoscopy shows mucosal erythema, edema, nodularity, erosions, and ulcerations [65, 66]. The causes of this condition are poorly clarified but are probably due to an imbalance in the colic microbiome and to the consequent decrease in the production of SCFAs. Indeed, an inverse correlation between the presence of *Bifidobacterium*, which is one of the principal producers of SCFAs, and the severity of diversion colitis has been documented [67]. Other consequences of the deficit of SCFAs are a possible increase in arteriolar resistance that leads to ischemia in the colon and a reduction in the production of mucin; both of these consequences may be related to the occurrence of diversion colitis [111]. Colic inflammation is also thought to be related to the relative prevalence of nitrate-reducing bacteria (NO producers) [112]. Medical therapy for the treatment of diversion colitis consists of SCFAs and 5-aminosalicylic acid (5-ASA) enemas, steroids or fiber irrigation [65, 66]. A recent trial demonstrated that preliminary anterograde stimulation of the efferent limb of the ileostomy and of the diverted colon in patients undergoing ileostomy reversal had very promising results in terms of shortened postoperative stay [113]. Moreover, autologous fecal transplantation, which showed promising results in the treatment of pouchitis, has also been proposed for the treatment of patients not manageable with alternative medical or surgical therapy [114, 115].

### Influence of perioperative management on microbiota: the role of ERAS protocols

Enhanced Recovery After Surgery (ERAS) protocols are a standardized and coordinated care pathway designed to improve the multidisciplinary management of surgical patients [36,^ 38^, 116] and reduce postoperative complications and hospital stay [117, 118]. ERAS protocols consist of various recommendations for perioperative management, including preoperative, intraoperative and postoperative measures. Preoperative measures include a preliminary phase, consisting of counseling and patient education on surgical complications, stoma handling, nutrition and bowel preparation. In the immediate preoperative phase, ERAS protocols recommend a clear liquid diet up to 2 h before general anesthesia, carbohydrate-rich beverage intake and the avoidance of mechanical bowel preparation (MBP) [38, 119]. Intraoperatively, ERAS protocols regulate the modality of anesthesia, the use of pain control agents, antiemetic prophylaxis, intraoperative fluid management and the surgical approach. Postoperatively, ERAS protocols are focused on early mobilization and feeding. The use of opioids during and after surgery is also highly discouraged due to the increased risks for POI and postoperative complications [120]. All these measures are in line with the principle of reducing surgical stress; therefore, the use of ERAS protocols has great theoretical potential to minimize changes in the GM [36]. Nevertheless, only one study so far has investigated the possible correlation between the application of ERAS measures and changes in GM composition. In 2016, Shakhsheer et al. investigated the role of morphine administration on the integrity of the intestinal anastomosis and on the composition of the GM in a murine model. They performed a 1-cm colic resection with rectosigmoid anastomosis. Postoperatively, rats had free access to water and food. The rats were divided into two groups: one with a subcutaneous pellet slowly releasing morphine and one releasing placebo. All rats were sacrificed, and the integrity of the anastomosis was assessed by the anastomotic healing score (AHS). An AHS of 0 meant normal healing of the anastomosis, and an AHS of 4 meant a perianastomotic abscess with evident leakage. Of the 30 placebo-treated rats, only 1 had an AHS of 3, while all the others had an AHS of 0. By contrast, 48.39% of the morphine-treated rats had an AHS of 0, 22.58% of them had an AHS of 1–2, 19.35% of them had an AHS of 3 and 9.48% an AHS of 4. The composition of the microbiota in the two groups was evaluated, highlighting how morphine-treated rats had an increase in *P. aeruginosa* and in the high-collagenase phenotype of *E. faecalis* at the anastomotic site. The authors also demonstrated how both endogenous and exogenous morphine could directly activate *E. faecalis* to produce collagenases and affect the chemoattractant and adhesive capacity of *P. aeruginosa* [121].

### The role of MBP and OAB preparations (Tables 2, 3)

**Table 2:** Meta-analyses investigating the role of MBP and OABs in elective colorectal surgery

Author (year)	Sample size	Design	Groups	Outcomes	Results	Quality assessment/risk of bias
Rollins (2019) [8]	Total: 69,517 patients; 28 RCTs with 6437 patients; 12 cohort studies with 63,080 patients	Meta-analysis of RCTs and non-randomized studies	1. MBP + OABs vs MBP 2. MBP + OABs vs OABs 3. MBP + OABs vs no preparation 4. OABs alone versus no preparation 5. OABs vs MBP	SSI, anastomotic leak, 30-day mortality, overall morbidity, development of ileus, reoperation and Cdiff infection	1.—SSIs reduced in the combined and in the RCT analysis - AL, mortality, morbidity, ileus, reduced in the combined but NS in the RCT analysis - Cdiff NS 2.—30 days mortality, ileus reduced in the combined but NS in the RCT analysis - other outcomes NS in the combined and RCT analysis 3. RCTs unavailable 4. RCTs unavailable 5. RCTs unavailable	Variable risk of bias in the RCTs, poor documentation on randomization methods, allocation concealment, and blinding Heterogeneity due to the different antibiotic regimens
Toh (2018) [9]	38 RCTs with 8458 patients	Network meta-analysis of RCTs	1. MBP + OABs vs MBP 2. MBP + OABs vs OABs 3. MBP + OABs vs no preparation (indirect comparison) 4. OABs alone versus no preparation (indirect comparison) 5. MBP vs no preparation 6. OAB vs MBP	Primary: total, incisional, and organ/space SSI Secondary: anastomotic leak, mortality, readmissions/reoperations, UTI, pulmonary complications	Primary: 1. Reduced total, incisional and organ/space SSIs 2. NS 3. Reduced total SSIs 4. Reduced organ/space SSIs 5. Reduced organ/space SSIs 6. SSIs NS Secondary: NS for every outcome and every comparison	Variable risk of bias, heterogeneity + + + for the different resection sites and antibiotic treatments, no RCTs in every category led to the use of indirect comparisons

**Table 3:** RCTs investigating the role of OABs in elective colorectal surgery

Author (year)	Trial Name/Type/Country	Status	Analyzed sample size and included population	Intervention	Outcome	Results
Abis (2019) [10]	SELECT *NCT01740947* *Phase III* *Multicentric* *Netherlands*	Stopped at interim analysis (superiority not attainable)	455 patients (762 planned) All colorectal resections	OABs (colistin, tobramycin and amphotericin B) vs no preparation MBP + OABs vs no preparation for left-sided and rectal resections	AL (primary outcome) Infectious complications (secondary outcome)	NS difference in AL (6.1 vs 9.7%, OR 0.61 CI 95%, 0.30–1.22) Reduced infectious complications (14.9 vs 26.9%, OR 0.47, CI 95% 0.29–0.76 at the multivariable analysis)
Koskenvuo (2019) [11]	MOBILE *NCT02652637* *Phase III* *Multicentric* *Finland*	Completed	396 patients All colorectal resections	MBP + OABs (neomycin and metronidazole) versus no bowel preparation	SSIs (primary outcome) AL, reoperation rate (secondary outcomes)	NS difference in SSIs (7 vs 11%, OR 1·65, 95% CI 0·80–3·40; p = 0·17), AL (4 vs 4%), reoperation rate (8 vs 7%)
Schardey (2020) [12]	*-* *-* *Phase III* *Monocentric* *Germany*	Stopped at interim analysis (ethical reasons)	80 patients (280 planned) Only rectal resections	MPB + OABs (polymyxin B, tobramycin, vancomycin, and amphotericin B) versus MBP + placebo (+ amphotericin B)	AL (primary outcome) Infectious complications (secondary outcome)	Reduced leak in the MPB + OABs group (5% versus 20%, p = 0.0425) NS difference for infectious complications
Basany(2020) [13]	ORALEV *NCT02505581* *Phase III multicentric* *Spain*	Completed	536 patients Only colic resections (no rectal resections)	OABs (ciprofloxacin + metronidazole) vs no OABs	SSIs	Reduced SSIs in the OAB group (5% vs 11%, p = 0.013). Organ/space SSIs similar (1.9% vs 2.6%, p = NS)
	ORALEV2 *NCT04161599* *Phase III multicentric* *International*	Recruiting	686 patients planned	MBP + OABs vs. OABs alone	SSIs	Ongoing

MBP has been a surgical dogma for more than a century. MBP was administered to patients undergoing elective colorectal surgery or other abdominal operations where entrance into the colonic lumen was anticipated as a possible event. This practice was based on the theoretical rationale that the mechanical removal of feces and associated microbes would result in a lower morbidity rate, particularly in fewer SSIs and ALs [122, 123].

MBP had unclear benefits and possible disadvantages, including patient discomfort; fluid and electrolyte imbalance; and alterations of the GM and of the colonic mucus layer, with possible increased bacterial translocation [18,^ 124^, 125]. The administration of MBP has been progressively abandoned after the accumulation of results on its lack of efficacy in numerous randomized controlled trials (RCTs) [126, 127], culminating in the publication of a 2011 Cochrane systematic review and meta-analysis that confirmed the absence of benefits in surgical outcomes associated with this practice [128]. Moreover, MBP was reported to actually increase the risk for postoperative SSIs in another meta-analysis [129].

The role of intravenous antibiotic prophylaxis was assessed in the early 1980s by Baum et al., and this technique was extensively applied in the following years [130]. In 2014, a Cochrane review identified a 65% risk reduction in patients receiving intravenous prophylaxis (relative risk (RR) 0.34, 95% confidence interval (CI) 0.28–0.41), p < 0.00001) [131], validating the role of intravenous prophylaxis, which is not currently being questioned. In recent years, based on the hypothesis of previous sporadic findings [132], it has been postulated that the association of MBP with selective decontamination of the gastrointestinal tract through the administration of nonabsorbable OAB preparations could be beneficial based on the rationale that MBP would reduce the fecal bulk and improve OAB delivery to the bowel mucosa [132]. Two large retrospective studies based on data from the American College of Surgeons National Surgical Quality Improvement Program (ASC-NSQIP) reported a significant role of MBP + OABs in reducing SSIs and overall postoperative complications [133, 134], and the guidelines of major American surgeon societies have changed accordingly [135–137].

Recent high-quality meta-analyses have also addressed this topic. In 2018, Rollins et al. conducted a comprehensive systematic review and meta-analysis of RCTs and observational cohort studies with the aim of comparing the association between colorectal surgery postoperative outcomes and the use of OABs with or without MBP [138]. According to the availability of the studies for each comparison, this group detected a significantly reduced risk of SSIs with the use of MBP + OABs compared with the use of MBP alone, and this finding remained consistent even when analyzing only the RCT results. No significant difference in SSIs for the comparison the use of MBP + OABs vs the use of OABs alone was found, neither in the combined analysis nor in RCTs. A significant reduction in SSIs for patients undergoing MBP + OABs vs no preparation and patients receiving OABs alone vs no preparation was found only in cohort studies, as RCTs were not available. Regarding AL, a reduced risk of AL was detected for the use of MBP + OABs vs the use of MBP alone only in the combined analysis, but this result was not confirmed in RCTs. No significant difference was detected for the use of MBP + OABs vs the use of OABs either in the combined analysis or in RCTs, and a significant reduction in AL was documented for the use of MBP + OABs vs no preparation only in cohort studies, as RCTs were not available. Interestingly, a subgroup analysis conducted in patients undergoing open and laparoscopic procedures documented that in the combined analysis, the benefit of the use of MBP + OABs vs the use of MBP was limited to open procedures. In 2018, Toh et al. [139] presented the results of a network meta-analysis of RCTs focused on the impact of the different bowel preparation regimens on SSIs. Their final results demonstrated a significant risk reduction in total and incisional SSIs with the use of MBP + OABs vs the use of MBP and a tendency towards reduced organ/space SSIs. Moreover, they detected a possible advantage for the use of MBP + OABs vs the use of OABs alone in the total rate of SSIs, even though this advantage was less clear in the analysis for incisional SSIs (where no difference between the use of MBP + OABs and the use of OABs was detected) and for organ/space SSIs (where the use of OABs alone ranked better than the use of MBP + OABs). The authors reported some limitations of the study due to the heterogeneity of the included RCTs in terms of disease location, the paucity of RCTs directly comparing the use of MBP + OAB vs no preparation and the use of OABs vs no preparation that permitted only indirect comparisons, and the paucity of RCTs reporting data on laparoscopic procedures.

In 2017, an international, multicenter, prospective audit was conducted by the European Society of Coloproctology Collaborating Group on patients undergoing left-sided resections only. This audit was focused on the association among MBP, OABs and AL [140]. Among 3676 patients followed prospectively, the group of 618 patients receiving preoperative MBP + OABs presented the lowest rate of AL (6.1%) when compared with patients receiving MBP (9.2%) and patients receiving no bowel preparation (8.7%). Patients undergoing MBP + OAB treatment had relatively favorable clinicopathological characteristics (fewer cardiovascular comorbidities, lower body mass index, lower rate of active smoking). Nevertheless, the risk of AL was almost halved in this category (odds ratio (OR) 0.52, 0.030–0.092, p = 0.02) after assessment by multivariable regression.

The safety and feasibility of OAB administration have already been investigated in some retrospective preliminary studies [141]. In 2019, the results of the SELECT multicenter RCT, which investigated the role of OABs in reducing the leak rate of patients undergoing colorectal surgery, were published [142]. In this trial, all locations of colectomy were included, but MBP was administered only for left-sided and rectal resections. A standardized ERAS protocol was applied for all patients. The results demonstrated a significant reduction in postoperative infectious complications in the OAB group (14.9 vs 26.9%, OR 0.47, 95% CI 0.29–0.76 in the multivariable analysis). However, the reduction in postoperative infectious complications was not the primary outcome of the trial, which was stopped after an interim analysis because it failed to demonstrate the superiority of the use of OABs in reducing the rate of AL (6.1 vs 9.7%, OR 0.61 95% CI, 0.30–1.22). The multicenter MOBILE RCT, also published in 2019, compared the use of MBP + OABs vs no bowel preparation to reduce the risk of SSIs. A perioperative ERAS protocol was applied, and all colorectal resections were included (rectal resections represented 2.5% of the trial population). The results did not show any significant difference in regard to SSIs, AL or reoperation rate between the two groups [143]. In 2020, the late report of an RCT conducted in 1999–2004 was published [144]. In this RCT, the use of MBP + OABs vs MBP was investigated in patients undergoing rectal surgery. This RCT was stopped at an interim analysis after the recruitment of only 80 patients (vs the 280 patients initially planned) due to a statistically significant reduction in the risk of AL in patients receiving MBP + OABs (5% vs 20%, p = 0.0425). Finally, the results of ORALEV, a phase III multicenter RCT comparing the use of OABs vs no OABs in patients undergoing colic resection, were recently published. This trial purposely excluded patients undergoing rectal resection. In the experimental arm, OAB therapy consisted of a one-day oral administration of ciprofloxacin and metronidazole. SSIs were the primary outcome, and the number of SSIs was significantly reduced in the OAB arm (5% vs 11%, p = 0.013) [145]. However, this reduction was limited to superficial/deep SSIs, as the number of organ/space SSIs was similar (1.9% vs 2.6%). Following the results of this study, the authors planned a second trial, ORALEV 2 (NCT04161599), that focused on the comparison between the use of MBP + OABs and the use of OABs alone, with SSIs as a primary outcome.

In conclusion, there is promising evidence on the role of MBP + OABs in reducing SSIs, especially in left-sided and rectal resections, where the use of MBP is widespread, and, therefore, this comparison of the use of MBP + OABs vs the use of MBP is more pertinent. Overall, OAB preparation, either in combination with MBP or alone, showed promising evidence in the prevention of postoperative complications after elective colorectal surgery. The use of OABs alone seemed to reduce the incidence of SSIs when compared with no preparation. For the comparison between the use of MBP + OABs vs the use of OABs alone or no preparation, more evidence is needed, especially in regards to right-sided resection, in which the risk of AL is lower, the use of MBP is not routinely applied, and the risk of SSIs has already been minimized by the widespread use of laparoscopy [146].

### The role of probiotics and synbiotics

In addition to the administration of topical antibiotics to reduce the number of pathogenic species in the GM, GM manipulation has also been attempted through the administration of probiotics, prebiotics and synbiotics, with the aim of obtaining a shift in the balance between nonpathogenic species and pathogenic species. Probiotics have been defined as “live microorganisms that, when administered in adequate amounts, confer a health benefit on the host” [147]. After surviving transit in the proximal gastrointestinal tract, the primary activity of probiotics occurs in the colon. By contrast, prebiotics have been defined as “nonviable food component that confer health benefit(s) on the host associated with modulation of the microbiota” [148]. Last, synbiotics have been defined as a combination of probiotics and prebiotics [149]. In previous studies, probiotics have shown numerous properties, including a strong anti-inflammatory effect and the capability of antagonizing the overgrowth of pathogenic bacteria (*P. aeruginosa*, *S. aureus*, *E. coli* and others) [150–152]. The most well-known and utilized probiotics are those of *Lactobacillus* and *Bifidobacterium* spp. [149]. Most studies have evaluated the possible benefits of probiotics when administered in the perioperative setting, with mixed results. In particular, some perplexity has arisen because the beneficial effect of probiotics was not consistent in all patients [153]. Recently, many comprehensive systematic reviews and/or meta-analyses have investigated the association between the administration of probiotics and synbiotics and abdominal surgery outcomes [154–156]. These systematic reviews and/or meta-analyses found only a few studies that targeted the sole use of prebiotic; therefore, they focused on probiotics and synbiotics, including 34 RCTs, in their analysis. Most of the included studies used multistrain probiotics. The results showed that the administration of probiotics and synbiotics almost halved the relative risk for postoperative infectious complications (RR 0.56, 95% CI 0.46–0.69, p < 0.00001), with an even greater effect in the subgroup of patients receiving synbiotics (RR 0.46, 95% CI 0.33–0.66, p < 0.00001). A positive trend was found for an increased duration in the administration of probiotics and synbiotics and diminished postoperative infectious complications, but the difference did not reach statistical significance. In the subgroup of patients receiving synbiotics, the postoperative length of stay was significantly reduced. The role of the administration of probiotics and synbiotics in patients undergoing colorectal surgery was analyzed by Darbandi et al. [155]. In their systematic review, they identified 21 relevant clinical trials and summarized that patients treated with probiotics and synbiotics had fewer postoperative infectious complications and SSIs and a shorter duration of hospital stay. The beneficial effects in terms of postoperative infections were imputed to a positive stimulation of innate immunity by the supplements. Darbandi et al. also reported that probiotics and synbiotics led to a reduction in intestinal permeability and bacterial translocation and to a greater preservation of the ratio of *Lactobacillus* spp. to *Bifidobacteria* and *Enterobacteriaceae* spp. Last, probiotics and synbiotics led to a lower grade of severe postoperative diarrhea and an increased postcolectomy quality of life in all gastrointestinal domains as assessed by validated questionnaires. Liu et al. conducted another meta-analysis in a population of patients undergoing colorectal surgery, including 9 RCTs, and confirmed that the use of multistrain probiotics was beneficial in reducing the total number of infections (OR = 0.30, 95% CI: 0.15–0.61, *p* = 0.0009), including both SSIs (OR = 0.48, 95% CI: 0.25–0.89, *p* = 0.02) and non-SSIs (OR = 0.36, 95% CI: 0.23–0.56, *p* < 0.00001). However, the use of non-multistrain probiotics did not reduce the number of total infections or SSIs [156].

No significant adverse effects for the administration of probiotics and synbiotics were documented in any of these reviews. Of note, probably due to the limitations of the included trials, these systematic reviews did not report on the eventual administration of MBP or OABs. In summary, the use of symbiotic and multistrain probiotics had a significant effect on reducing the incidence of postoperative infectious complications, the length of stay and gastrointestinal symptoms after abdominal surgery, particularly colorectal surgery.

### Considerations for future research

Growing evidence supports the role of the GM as an influencer of colorectal surgery outcomes. To date, studies have been limited by difficulty in the isolation of the different GM species due to the scarce availability and increased cost associated with sequencing techniques and animal laboratories. Most of the in vivo studies conducted so far have been performed on animal models, which has partially limited the clinical application of the findings. Indeed, contrary to manipulated murine species, in humans, there is nonnegligible interpersonal variability in the composition of the GM. A solution to the problem of interpersonal variability would be the development of specific “signatures” related to the different colorectal surgery outcomes. A useful strategy to further understand the GM could be the use of artificial intelligence (AI) strategies to process the large amount of genomic information and develop the “signatures” through machine-learning strategies. From a clinical point of view, it seems reasonable to conduct further studies that associate individual pathogen species or different GM compositions with the development of complications and to systematically test the composition of preoperative and postoperative microbiota to identify shifts in its composition or virulent shifts in individual species. Other studies should assess the clinical relevance of the manipulation of the GM through different antibiotics and synbiotics. All these findings should be weighted on the new use of minimally invasive techniques.

## Conclusions

The role of the GM in outcomes following colorectal surgery is becoming increasingly apparent. There is an emerging need to implement translational research on the topic, to confirm the animal results in humans and to correctly assess the magnitude of influence of the GM and its contribution to the multifactorial occurrence of postoperative infective complications. Future studies should clarify the preoperative and postoperative composition of the GM and the impact of the GM on the different colorectal surgery complications and assess the validity of GM-targeted measures in effectively reducing these events. Current evidence promotes the application of ERAS protocols and of all measures aimed at reducing surgical stress. Current evidence also validates the preoperative administration of multistrain synbiotics and probiotics. In regard to the direct manipulation of the GM with MBP ± OABs, this strategy is still under investigation in right-sided colic resection but is very promising for left-sided colic resection, where MBP and OABs seem capable of significantly reducing the occurrence of SSIs and, possibly, AL.

## Data Availability

The datasets used and/or analysed during the current study available from the corresponding author on reasonable request.

## Abbreviations

GM: Gut microbiota
ERAS: Enhanced recovery after surgery
SSI: Surgical-site infection
MAM: Mucosal-associated microbiota
LM: Luminal microbiota
SCFA: Short-chain fatty acids
EHEC: Enterohaemorrhagic *Escherichia coli*
GF: Germ-free
MGB: Microbiota-gut-brain
POI: Postoperative ileus
AL: Anastomotic leak
ROS: Reactive oxygen species
AHS: Anastomotic healing score
MBP: Mechanical bowel preparation
OABs: Oral antibiotics
AI: Artificial intelligence
